# Induction and repression effects on CYP and transporter protein abundance by azole mixture uptake in rat liver

**DOI:** 10.17179/excli2020-2311

**Published:** 2020-06-26

**Authors:** Helen Hammer, Flavia Schmidt, Tanja Heise, Constanze Knebel, Alexander Dabrowski, Hannes Planatscher, Carsten Kneuer, Philip Marx-Stoelting, Oliver Pötz

**Affiliations:** 1 NMI Natural and Medical Sciences Institute at the University of Tübingen, Reutlingen, Germany; 2 SIGNATOPE GmbH, Reutlingen, Germany; 3 BfR, Department of Pesticides Safety, Max-Dohrn-Str. 8-10, 10589 Berlin, Germany

**Keywords:** ABC transporter, azole fungicides, cytochrome P450, Immunoaffinity-based mass spectrometry, liver toxicity, pesticides, pesticide mixtures, SLC transporter

## Abstract

Detection of mixture effects is a major challenge in current experimental and regulatory toxicology. Robust markers are needed that are easy to quantify and responsive to chemical stressors in a broad dose range. Several hepatic enzymes and proteins related to drug metabolism like cytochrome-P-450 (CYP) enzymes and transporters have been shown to be responsive to pesticide active substances in a broad dose range and are therefore good candidates to be used as markers for mixture toxicity. Even though they can be well quantified at the mRNA level, quantification on the protein level is challenging because most of these proteins are membrane bound. Here we report the development of mass spectrometry-based assays using triple-x-proteomics (TXP) antibodies in combination with targeted selected ion monitoring (tSIM) to quantify changes of protein levels due to exposure to mixtures of pesticide active substances. Our results indicate that changes on the protein level of CYP1A1, ABCB2, ABCC3 are in line with observations on the mRNA and enzyme activity level and are indicative of mixture effects. Therefore, the tests are promising to reveal effects by chemical mixture effects in toxicological studies in rats.

## Abbreviations

ABC, ATP Binding cassette; CYP, Cytochrome P450; EN peptide, endogenous peptide; IS peptide, C^13^/N^15^ labeled peptide; SISCAPA, stable isotope standards and capture by anti-peptide antibodies; SRM, single reaction monitoring; tSIM, targeted selected ion monitoring; TXP, triple-x-proteomics

## Introduction

Detection of mixture effects is a major challenge in current experimental and regulatory toxicology (OECD, 2018[21]). Robust markers are needed that are easy to quantify and responsive to chemical stressors in a broad dose range. Several hepatic enzymes and proteins related to xenobiotic metabolism like cytochrome-P-450 (CYP) enzymes and transporters have been shown to be responsive to pesticide active substances in a broad dose range (Heise et al., 2015[12], 2018[11]; Schmidt et al., 2016[25]) and are therefore good candidates to be used as markers for mixture toxicity. Even though the transcripts can well be quantified at the mRNA level, quantification on the protein level is challenging because most of these proteins are membrane associated. Nevertheless, quantification at the protein level is regarded important to verify functional relevance of effects detected at mRNA level and to come to sound regulatory conclusions (Marx-Stoelting et al., 2015[18]).

Within the last two decades, the quantification of peptides in enzymatically digested biological samples as protein surrogates using targeted mass spectrometry approaches has been widely established (Gerber et al., 2003[10]). By the introduction of stable isotope labelled peptides (IS) as internal standards, an indirect quantification of proteins by mass spectrometry was achieved. A hybrid technology termed stable isotope standards and capture by anti-peptide antibodies (SISCAPA) (Anderson et al., 2004[1]) or immuno-single reaction monitoring (Immuno-SRM) (Whiteaker et al., 2011[33]) combined immunoaffinity precipitation with targeted mass spectrometry read out. This hybrid approach allows the analysis of large sample sets and improves sensitivity compared to mass spectrometry-based assays alone. Motif-specific antibodies also termed TXP antibodies comprise short C-terminal epitopes of four amino acids (Poetz et al., 2009[24]). TXP epitopes address dozens to hundreds of peptides of a digested proteome thereby allowing the enrichment of peptide groups (Planatscher et al., 2010[22]), since the epitopes are comprised in hundreds of peptides. This antibody feature can be utilized to overcome the challenge of the availability of antibodies needed to cover the human proteome (Anderson et al., 2009[2]) For example, while coverage of the proteome by Immuno-SRM would require more than 70,000 sequence-specific antibodies (Anderson et al., 2009[2]), the use of TXP antibodies reduces the need for antibodies by more than tenfold (Planatscher et al, 2010[22]). With regard to specificity and cross-reactivity, a different concept has to be applied to characterize the antibodies' quality (Planatscher et al., 2014[23]; Uhlen et al., 2016[27]). Since the epitope of TXP antibodies does not perfectly match the antigen used, but is rather an epitope motif, the peptide groups that can be enriched with each antibody must be carefully determined (Planatscher et al., 2014[23]).

The TXP approach has the potential to be used in discovery experiments, but has so far only been used for specific protein analyses. Therefore, the antigens for immunization are selected in such a way that they are able to cover a panel of proteins of interest with the smallest possible number of antibodies (Planatscher et al., 2010[22]). The concept is very efficient for protein families such as cytochrome P450 enzymes and G-protein coupled receptors (Eisen et al., 2013[8]; Weiß, 2015[29]; Weiß et al., 2015[31]). By selecting proteotypic peptides with highly conserved C-termini, the peptides can be immunoprecipitated and the proteins can be identified and quantified by LC-MS with a relatively small number of antibodies (Weiß, 2015[29]; Weiß et al., 2015[31]; Hoeppe et al., 2011[13]). In earlier studies it was demonstrated that the introduction of an immunoprecipitation step using TXP antibodies improved sensitivity of LC-SRM (Weiß et al., 2015[31]) or LC-tSIM-based protein assays prior mass spectrometric read out (Weiß, 2015[29]).

The method can be easily applied to other sample matrices like urine, plasma, serum, tissues and cell culture material after minor adaptions (Weiß, 2015[29]; Gautier et al., 2016[9]). For non-fluid samples like tissues or cell cultures, proteins must be extracted before the enzymatic fragmentation. The challenge here is to identify the most efficient solubilization method which provides the best results for the protein panel of interest, e.g. for membrane proteins for instance. The following steps of the process are largely identical, regardless of which sample is analyzed: First, proteins are enzymatically fragmented using trypsin, second, C^13^/N^15^ labeled peptides (IS peptides) are added for quantification, third, TXP antibodies are applied in a immunoprecipitation step, fourth, enriched peptides are separated by reverse phase chromatography and finally, peptides are detected and quantified by LC-MS. Since the EN peptides derived from the endogenous proteins of interest and the synthetic IS peptide standards do not differ in their physicochemical properties but the mass, EN and IS peptide pairs are enriched stochiometrically and co-eluted in the chromatography step. Hence, the use of IS peptides in the workflow compensates for losses in immunoprecipitation efficiency, chromatography and ion suppression (Weiß et al., 2015[31]).

Azoles fungicides target a fungal enzyme (CYP51) involved in membrane biosynthesis. This pesticide group, which is widely used in agriculture, was selected by EFSA as model substances to study cumulative effects (EFSA, 2009[7]). The main effects of azoles in mammals can be observed in the liver. However, developmental toxicity has also been shown (EFSA, 2009[7]). Administration of cyproconazole, epoxiconazole (EFSA, 2010[4]; 2008[6]), prochloraz (EFSA, 2011[5]) and mixtures thereof to rats and mice induce liver atrophy and enzymes (Heise et al., 2018[11]). In earlier studies in rats, we confirmed that interaction of azoles with the nuclear receptors CAR, PXR and partially also AhR led to hepatotoxicity (Heise et al., 2015[12], 2018[11]; Schmidt et al., 2016[25]). As a consequence, changes in CYP-enzyme and transporter mRNA expression were observed. Here we report the development of a method for detection of such changes on the protein level and the application to an animal study to investigate the effect of fungicides and combinations thereof. 

## Methods

### Animal experiments

A 28-day feeding study in rats was conducted as described before (Heise et al., 2015[12]; Schmidt et al., 2016[25]). In short at the beginning of the study, healthy 8 to 9- week-old male Wistar rats (Crl/Wi, Charles River Wiga GmBH, Sulzfeld, Germany) were caged in groups of 5 and fed with phytoestrogen-free rodent standard diet (R/M-H V155, Ssniff, Soest, Germany) and filtered tap water ad libitum. The test substances were added to the feed. Housing and environmental conditions were in accordance with standard operation procedures of the test facility in the German Federal Institute for Risk Assessment. Detailed overview of dosages and groups has been described earlier (Heise et al., 2015[12]; Schmidt et al., 2016[25]) and can be found in the supplemental material (Supplementary Table 2). Test substances were administered in five dose levels to groups of 5 animals. A group of the same size obtained phenobarbital to serve as positive control (Sigma-Aldrich, Hamburg, Germany) and three negative control groups were fed pesticides-free diet. In a consecutive experiment, combinations thereof were used: the binary mixture contained cyproconazole and epoxiconazole and the ternary mixture cyproconazole, epoxiconazole and prochloraz. Groups of 10 rats were exposed to three different pesticide levels. Four control groups of five animals each were included in the experiments, two for each mixture. Animals were checked daily for clinical signs and mortality. After 28 days of treatment rats were deeply anesthetized with Sevofluran (Abbot, Germany) and finally killed in 95 % CO_2_ and 5 % O_2_. An accredited animal pathologist performed the necropsy and isolated the livers. The organs were briefly examined and weighed. The organs were splitted into smaller pieces and frozen in liquid nitrogen. Parts of the samples were used previously for mRNA expression experiments (Heise et al., 2015[12]; Schmidt et al., 2016[25]). The protein analysis described here, was only performed for the groups treated with NOAEL and 10xNOAEL cyproconazole, epoxiconazole, prochloraz and the mixtures thereof, as well as the according control groups.

### Test substances

Cyproconazole (Syngenta, Basel, Switzerland), epoxiconazole (BASF, Ludwigshafen, Germany), and prochloraz (BASF, Ludwigshafen, Germany) were obtained from the producers in the same quality and purity of which the substances are used in the final pesticide products. The pesticides were added to the rodent diet at five concentration levels by a solvent-free procedure by Ssniff (Soest, Germany).

The dose levels were chosen based on regulatory studies described in the approval procedure of the individual substance and ranged from a dose equivalent to a typical toxicological threshold level (NOAEL/100) till a dose level supposed to show toxic effects (10xNOAEL). The integrity and concentration of the test pesticides was controlled as described earlier (Heise et al., 2015[12]). The control diet was also analyzed to proof the absence of azole fungicides and to guarantee the quality of the negative control. 

### Protein quantification via the TXP methodology

CYP enzyme and transporter expression levels in the liver were determined using an immunoprecipitation workflow, which has been described previously (Wegler et al., 2017[28]; Weiß et al., 2018[30]). In short, liver tissue samples were homogenized using a ball mill. Subsequently, the powder was incubated with the 20-fold volume (µL) of the weighed tissue amount (mg) for one hour at room temperature under continuous rotation. In case of cell culture samples, the lysis buffer was added directly to the cell pellet. The protein concentration in the lysate was determined via a BCA assay (Pierce BCA Assay Protein Kit Thermo Scientific, Waltham, USA). 400 µg protein was enzymatically digested for 16 h using Trypsin (Pierce Trypsin Protease, MS-Grade Thermo Scientific, Waltham, USA) in a 1:20 ratio. Next, stable isotope labeled peptides and TXP antibodies (Pineda Antikörper-Service, Berlin, Germany) were mixed with an amount of 10 - 40 µg of digested protein, followed by a one hour incubation at RT. Protein G-coated magnetic beads (Invitrogen) were applied to capture the antibody-peptide complexes. A magnetic particle processor (KingFisher 96, Thermo Scientific, Waltham, USA) was used to wash the beads. Finally, peptides were eluted with formic acid and detected and quantified by LC-MS (UltiMate 3000 RSLCnano and tSIM - QExactive Plus™ Thermo Scientific, Waltham, USA) as described previously (Weiß et al., 2018[30]). Data analysis was performed using Pinpoint 1.4 (Thermo Scientific, Waltham, USA). Peak areas of internal peptide standards and signals derived from the endogenous proteins were used to form ratios: isotopically-labeled peptide standard: endogenous peptide. The known amounts peptide standards were used to calculate endogenous amount of the respective surrogate peptides. 

### Epitope-motif analysis of antibodies

The epitope motifs were determined as described previously (Planatscher et al., 2014[23]; Weiß et al., 2018[30]). In short, a human cell line blend containing equal amounts of HepG2, HEK 293 and HCT116 was prepared as described above. The immunoprecipitation was performed with 5 µg antibody from 20 µg sample. The precipitated peptides were analyzed with a previously described non-targeted MS method (Weiß et al., 2018[30]). The peptide identification was performed with Proteome Discoverer 1.3 and Mascot and SEQUEST algorithms using UniProt Homo Sapiens reference proteome (June 2014). To create the sequence logo, (WebLogo 3, http://weblogo.threeplusone.com/create.cgi), the identifications of triplicates were merged and duplicate sequences removed. Unspecific binding was determined by using a monoclonal protein specific antibody with and without protein G beads. Peptides identified here, were considered as background and deleted. The four c-terminal amino acids were considered for the epitope sequence motif. For each identified c-terminal tag, the number of different peptide sequences was counted. All tags which were significantly enriched in comparison to an *in silico* digest, were included in the sequence logo.

### Dynamic range of MS-based immunoassays

A serial dilution of synthetic non-labelled peptides of a multiplexed assay were prepared in ELISA blocking reagent (Roche, Basel, Switzerland) and quantified in relation to a constant amount of isotopically labeled peptide. The EN peptides' recovery was calculated in percent of peptide amount which was used for the spiked-in. The dynamic range was defined as the range, where the standard deviation was lower than 20 % and the recovery between 80 and 120 %. The limits were set as lower and upper limits of quantification (LLOQ and ULOQ).

### mRNA expression and enzyme activity data

mRNA and enzyme activity data, which was compared to protein data, has been generated previously by Heise et al. (2015[12]). In case of the CYP enzymes, only data for the single substance treatment was available.

### Statistics

For the statistical analysis of the protein abundance data, a Student's-t-test (two tailed for heteroscedastic data using Excel 2016) was performed and Bonferroni's correction for multiple testing was applied. 

## Results

To analyze CYP and transporter protein expression in rat, 22 immunoaffinity mass spectrometry-based assays for the following proteins were developed: ABCB1, ABCB1a, ABCB11, ABCC2, ABCC3, CYP1A1, CYP1A2 , CYP2A1, CYP2A2, CYP2B2, CYP2B3, CYP2C11, CYP2C12, CYP2C13, CYP2C55, CYP2D3, CYP2E1, CYP3A9, CYP3A18, SLC10A1, SLC22A7, and SLC22A8. See Supplementary Table 1 for details on target proteins and protein surrogate peptides. Method development included characterization of epitope motifs, precision, accuracy and proteolysis kinetics of the surrogate peptides used for quantification.

### Epitope-motif analysis of antibodies 

**Figure 1:**
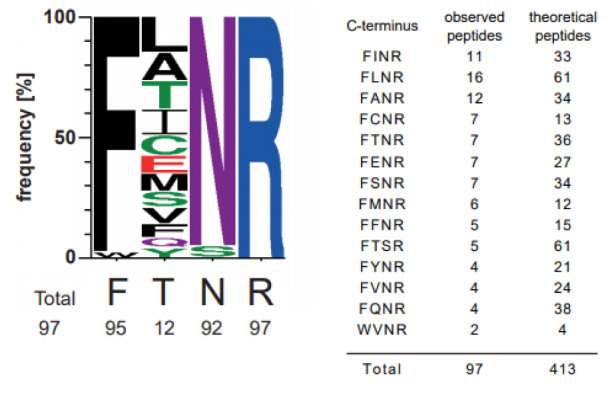
Characterization of anti-FTNR TXP antibody binding motif. Therefore a human cell line blend containing equal amounts of HepG2, HEK 293 and HCT116 was proteolyzed. The immunoprecipitation was done with 20 µg protein and subsequently, the precipitated peptides were identified via non-targeted MS-analysis. Based on UniprotKB (human ref. prot. June 2014), it was analyzed which c-termini were enriched significantly by the antibody and depicted as sequence logo. It is based on 14 c-termini which were observed in 97 peptides. Additionally, the number of tryptic peptides comprising these c-termini is given.

TXP antibodies have been generated towards four c-terminal amino acids of a peptide, e.g. FTNR. To analyze the actual binding-motif, the c-terminal tags of the precipitated peptides from the cell culture blend were analyzed and counted in how many different peptides each tag was present. The anti- FTNR antibody enriched 97 tryptic peptides with 14 different tags. It also enriched 19.9 % of all possible FTNR peptides determined by an *in silico* digest. The analyses of the peptide c-termini revealed that the antibody was highly specific in three of the four positions (Figure 1(Fig. 1)). At the second position the peptides showed a high variability, as twelve different amino acids were observed here. All antibodies selected for the assays enriched between 36 and 126 different peptides and between 8.6 and 33.3% of the peptides contained the exact C-terminus used for immunization (motifs are shown in Supplementary Figure 1).

### Assay precision and accuracy

**Figure 2:**
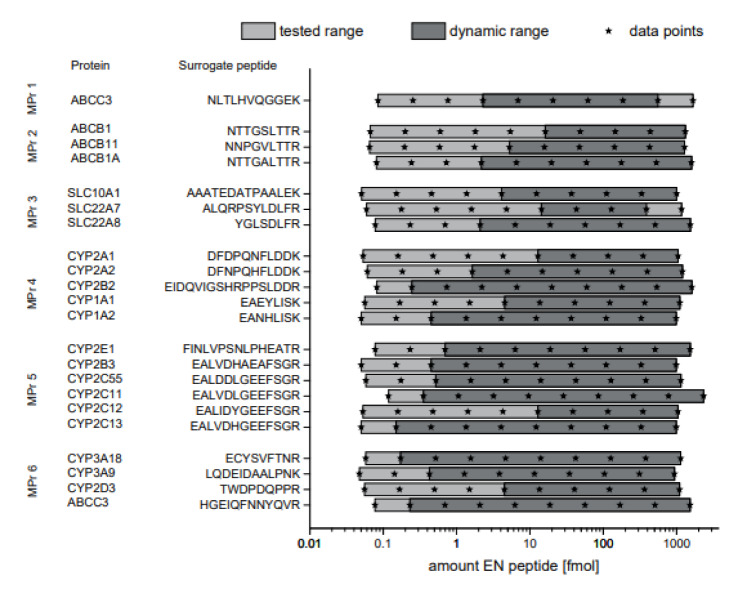
Dynamic range of MS-based immunoassays. Synthetic non-labeled peptides were serially diluted in artificial matrix and a constant amount of isotopically labelled peptides. The recovery of the non-labeled peptide in % and relative standard deviation are plotted. 80 and 120 % recovery are indicated with lines. The dynamic range of the assay was defined as the range in which the recovery was between 80-120 % and RSD below 20 %. Gray boxes indicate the range of the analyte in the rat liver samples. n=3

The assay precision and accuracy were determined between 0.05 fmol and 1600 fmol peptide using digested fish gelatin as surrogate matrix (Elisa Blocking Reagent, Roche). The dynamic range was determined according to the following criteria: 80-120 % recovery and less than 20 % relative standard deviation. The limits of the dynamic range were set as lower limit of quantification (LLOQ) and upper limit of quantification (ULOQ). The assays showed dynamic ranges between one and four orders of magnitude. In most cases, the ULOQ was beyond the tested concentration range. Hence the highest tested amount was indicated as the ULOQ. The values for the LLOQ ranged between 0.15 fmol and 16 fmol peptide (see Figure 2(Fig. 2)).

### Tryptic proteolysis kinetics

**Figure 3:**
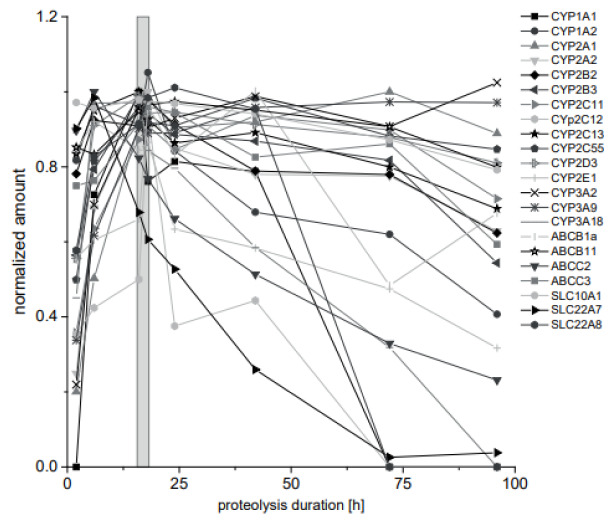
Kinetics of tryptic proteolysis of CYP and transporter proteins. Phenobarbital treated rat liver tissue from a 28-day feeding study was lysed and enzymatically proteolyzed. The kinetics of the proteolysis was monitored for 96 h. Results were normalized to the highest quantified amount for each analyte. The proteolysis duration, which is best to analyze all analytes from the same preparation, is indicated as gray box. Mean is given. Error bars are not shown for clarity.(n=3, 72 h and 96 h, n=1)

The protein quantification here and in other proteomics approaches is based on quantifying an endogenous proteotypic peptide as a protein surrogate. This peptide is released from the protein of interest by tryptic proteolysis and it is crucial to investigate the proteolysis efficiency. Hence, the kinetics of the peptide release was analyzed for each target protein. Additionally, the respective peptides with one N-terminal missed cleavage were monitored with exception of peptides greater than 25 amino acids. For 20 surrogate peptides, the missed cleavage variants could not be detected at any time point. For two surrogate peptides, the missed cleavage variant was detected at early time points. The proteolysis duration until maximal peptide release ranged between 2 h and 42 h. Seven peptides remained on a stable plateau with at least 80 % of the maximal amount for 96 h. The overnight proteolysis (16 h) was chosen as a compromise with regard to multiplex the highest number of analyses with a maximum in peptide release and is highlighted as box in Figure 3(Fig. 3).

### Hepatic CYP and transporter protein abundance in rats after fungicide treatment

**Figure 4:**
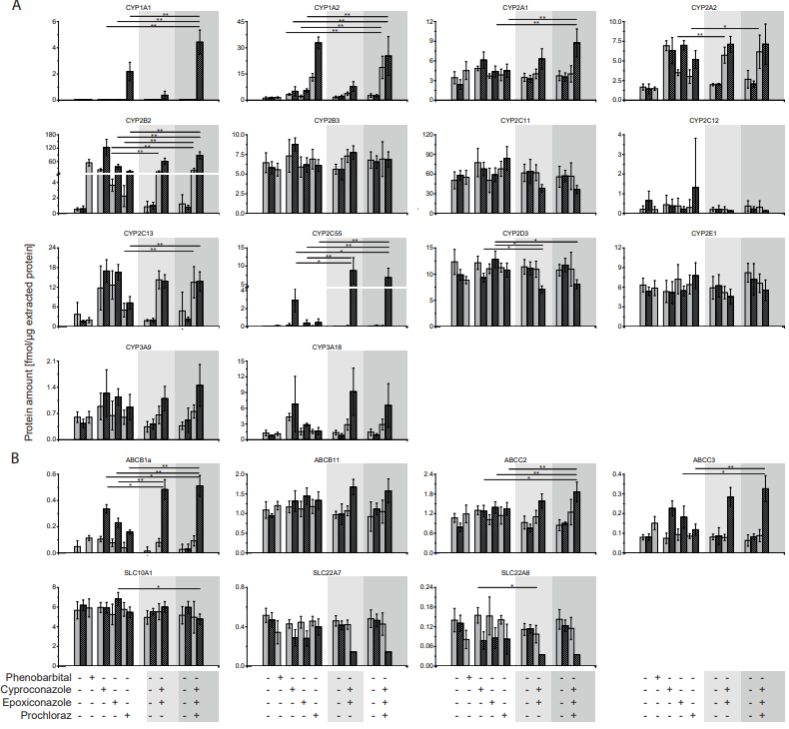
CYP and transporter protein abundance in livers of fungicide-treated rats. A 28-day feeding study in rats was conducted with cyproconazole, epoxiconazole and prochloraz as well as phenobarbital as positive control. Two doses were administered corresponding to NOAEL (gray bars) or 10x NOAEL (striped gray bars) based on a NOAEL derived from a regulatory guideline study. In a consecutive experiment, combinations thereof were used (mixture I: cyproconazole + epoxiconazole and mixture II: cyproconazole + epoxiconazole + prochloraz). (A) CYP and (B) transporter protein abundance in liver tissue was determined as fmol per µg extracted protein. Values below LLOQ were set to 0.5 LLOQ for further analysis. Mean and SD are given. Significant differences between mixtures and single substance treatment are indicated: * p<0.00238; ** p<0.00047. Control groups did not differ significantly. Control and single substance groups: n=5, mixtures: n=10

We applied the assays to a rat study which investigated the effects of three azole fungicides, cyproconazole, epoxiconazole and prochloraz, as well as binary and ternary combinations thereof (see Figure 4(Fig. 4) and Supplementary Table 2). Cyproconazole and epoxiconalzole were tested as binary mixture and cyproconazole, epoxiconazole and prochloraz as ternary combination. Additionally, the CAR activator phenobarbital was used as positive control. The studies with single substances have been conducted first and combinatorial treatments as a follow-up study. Hence, samples from a respective control group were analyzed for each study, six in total. None of the analyzed proteins differed significantly in protein abundance between the control groups (Supplementary Table 3E). 

The prototypical inducer Phenobarbital affected a 100-fold increase in CYP2B2 protein expression and a 130-fold increase of ABCB1a in the liver of the treated animals compared to the respective control group. No other CYP enzyme or transporter was observed to be increased by the phenobarbital-control group. Neither the protein abundance of a CYP1A or CYP3A-family member was increased as described in earlier publications for Wistar rats (Kishida et al., 2008[15]). However earlier publications were based on mRNA analysis and qPCR method. 

With regard to the single fungicide treatments CYP1A1 protein expression was elevated only at the high prochloraz dose - 10X NOAEL - whereas CYP1A2 was observed to be increased by both dosages - NOAEL and 10X NOAEL - of cyproconazole, epoxiconazole and prochloraz in the livers from the treated animals. In case of CYP1A1, the administration of the mixtures at high dose resulted in an induction of protein abundance to a larger extent compared to cyproconazole, epoxiconazole and prochloraz single treatment. Since the dosages of the fungicides were additively mixed in the combinatorial treatment, a non-additive effect was observed here for the binary and ternary mixture. Whereas in case of CYP1A2, the protein analyses revealed significantly stronger effects of both dosages using the ternary mixture. The CYP1A2 protein abundance was significantly higher than in the livers of the animals treated with single substances cyproconazole and epoxiconazole, but not higher than the result for prochloraz. Hence, the two additional fungicides cyproconazole and epoxiconazole did not result in a stronger effect with regard to liver CYP1A2 protein abundance which was dominated by prochloraz.

A slight protein induction by the ternary mixture was observed for the CYP family member of 2A1 compared to cyproconazole, epoxiconazole and prochloraz alone. In case of 2A2 a strong induction was observed by administration of cyproconazole at both doses, epoxiconazole and prochloraz at high dose as well as by both mixtures. Significant differences between combinatorial and single treatments were only observed for the low dose of the binary mixture with reference to epoxiconazole and the low dose of the ternary mixture in comparison to prochloraz. CYP2B2 protein abundance was found to be strongly induced by all three fungicides under single regime. Both combinatorial treatments induced CYP2B2 as strong as the strongest single substance cyproconazole. CYP2B3, CYP2C11 and CYP2C12 protein abundance was not found to be elevated after the fungicide treatment. In contrast CYP2C13 protein abundance increased up to 10-fold by fungicide dosing. A comparison of single versus combinatorial treatment revealed significant higher results for the combinatorial treatment with the ternary mixture than for prochloraz alone. Here a cyproconazole and epoxiconazole dominated effect can be observed. Most notably for the Cyp2c-subfamily, Cyp2c55 protein abundance was much stronger induced by the combinatorial treatments. It was increased up to 1000-fold by the high dose of the binary mixture and 90-fold by the ternary mixture in comparison to the respective control and 2 to 3-fold with respect to cyproconazole single treatment. Interestingly, the low dose did not show to have an impact on the Cyp2c55 protein expression regardless if single substances or combinations have been used. Also, the effect of single treatments resulted only in minor changes compared to the high dose of cyproconazole. Cyp2d3 protein abundance was hardly affected by the single treatments, whereas 10x NOAEL dose of both mixes decreased the protein abundance level significantly.

The two members of the Cyp3a family- 9 and 18 - were both induced by the fungicides compared to the controls. But the effects of the two combinatorial treatments were not significantly higher than the effect observed for the single substances. 

Compared to the CYP-enzymes the transporter protein abundances were affected to a lesser extent. ABCB1a was one of three transporters which were significantly changed on protein level by single and combinatorial treatments. Observed effects were stronger for the high dose combinations. Effects were additive but not higher than the summed abundance of ABCB1a transporter. In case of ABCB11 a protein increase compared to the controls was only observed for the high dose of the binary and ternary combinations but not for the single substances. ABCC2 was induced significantly higher by the high dose ternary treatment than by respective single substance treatments. Like for ABCB1a, ABCC3 protein abundance was elevated by high doses of the binary and ternary fungicide combination. The effect was significantly higher than observed with the epoxiconazole and prochloraz single treatments, but not higher than the effect by the cyproconazole. The three transporter SLC10A1, SLC22A7 and SLC22A8 showed no elevation in protein abundance induced by the fungicides. However, in case of SLC22A7 and SLC22A8 a reduction of the protein abundance resulted from the fungicide treatment. Most notably, the binary and ternary mixture reduced the protein abundance of SLC22A8 3-fold using the fungicides at the 10x NOAEL level.

### Comparison of protein abundance to mRNA expression levels and enzyme activity

**Figure 5:**
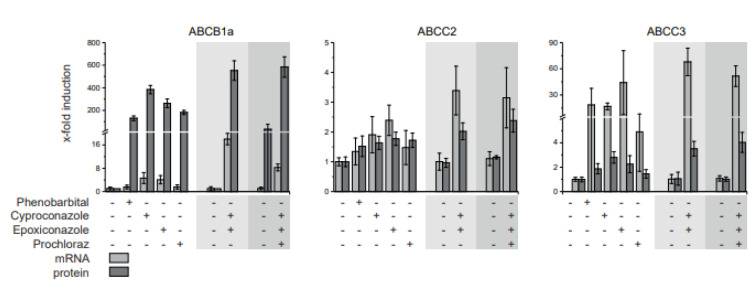
Comparison of transporter mRNA expression and protein abundance in livers of fungicide treated rats. A 28-day feeding study in rats was conducted with cyproconazole, epoxiconazole and prochloraz as well as phenobarbital as positive control. The dose corresponding to 10x NOAEL based on a NOAEL derived from a regulatory guideline study, was used to compare mRNA expression to protein abundance in liver tissue. Mean and SD are given. Control and single substance groups: n=5, mixtures: n=10

The transporters ABCB1a, ABCC2 and ABCC3 were analyzed further by comparing protein abundance to mRNA expression (Figure 5(Fig. 5)). Generally, the treatment influenced the transporters accordingly on protein as well as mRNA level. While the factor of induction of ABCC2 mRNA and protein is in the same range, ABCB1a protein abundance is increased up 100-fold as strong as mRNA expression. In case of ABCC3, the mRNA expression is induced 20-fold as strong as the protein abundance.

**Figure 6:**
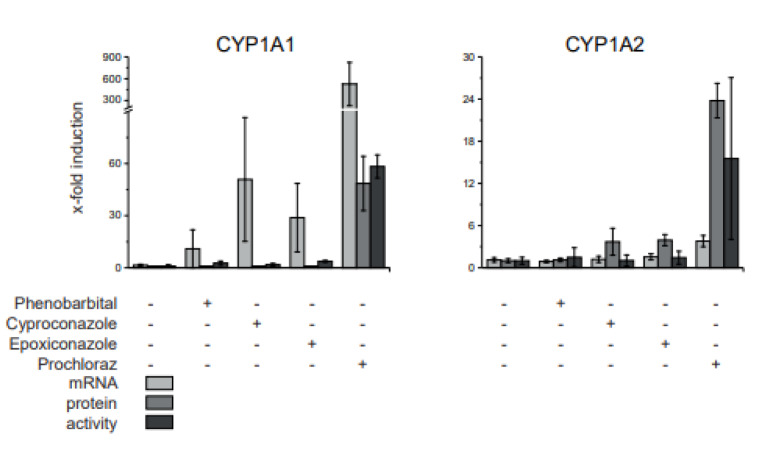
Comparison of CYP mRNA expression, protein abundance and activity in livers of fungicide treated rats. A 28-day feeding study in rats was conducted with cyproconazole, epoxiconazole and prochloraz as well as phenobarbital as positive control. The dose corresponding to 10x NOAEL based on a NOAEL derived from a regulatory guideline study was analyzed here. The protein abundance was compared to mRNA expression determined by qPCR and CYP activity determined by EROD (CYP1A1) and MROD (CYP1A2) dealkylation data published by Heise et al. (2015). The protein amount is expressed as fold change to control to compare it to mRNA expression and enzyme activity. Mean and SD are given. n=5

In case of the CYP enzymes, protein abundance was compared to mRNA expression and enzyme activity data in a reduced sample set: control, phenobarbital and single substances at 10xNOAEL (Figure 6(Fig. 6)). CYP1A1 and CYP1A2 protein and activity fold-induction was comparable. mRNA expression levels, on the other hand, differed. CYP1A1 mRNA is induced stronger than either protein or enzyme activity, while CYP1A2 mRNA expression is induced but not as dramatically as either protein abundance or enzyme activity.

## Discussion

In summary our results suggest that all three fungicides applied as single substances induced the expression of CAR and PXR targets, such as CYP2B2, CYP2C55 and ABCB1a in rat (Heise et al., 2015[12]; Martignoni et al., 2006[17]; Chen and Goldstein, 2009[3]). Additionally, the high cyproconazole dose reduced SLC22A7 (OAT2) protein expression, which was observed earlier on mRNA level and confirms CAR and PXR activation as previously described (Jigorel et al., 2006[14]). Moreover, only prochloraz induced CYP1A1 and CYP1A2 protein expression which are both target genes of AhR. The induction of these proteins are in line with mRNA expression data and enzyme activity suggesting that cyproconazole and epoxiconazole are CAR/PXR agonists and prochloraz is an agonist of all three nuclear receptors (Heise et al., 2015[12]; Marx-Stoelting et al., 2017[19]) also reviewed in Marx-Stoelting et al. (2020[20]). 

The strongest impact on protein level with regard to mixture effects was observed for CYP1A1 and CYP2C55. Whereas 1A1 is regulated by AhR, the corresponding human CYP2C55 analogues CYP2C8, 2C9 and 2C19 are regulated by CAR and PXR (Chen et al., 2009[3]). In case of the protein CYP1A1, the induction effect of prochloraz was twice as high when applied as ternary mixture compared to administration as single substance. Interestingly, the binary mixture of cyproconazole and propiconazole did also show an induction effect on CYP1A1 protein expression whereas in the animals treated with single substances no effect was observed. Similar observations were made in case of CYP2C55. The administration of the three fungicides alone led to an increase of CYP2C55 protein expression, but when applied as binary or ternary mixture, the effects where higher. The results observed are stronger than just additive as the data for ABCB1a suggests. For SLC22A7 and SLCA22A8, also termed OAT2 and OAT3, a mixture effect with regard to repression was observed. However, if this effect is synergistic or additive could not be determined due to a limited number of effect dose levels. Currently, more data needs to be collected to propose if a fungicide has a synergistic or additive effect on these proteins as well as clear criteria for when an effect has to be regarded as synergistic (Lasch et al., 2020[16]). Nevertheless, the assays developed here are suitable to be used for the analysis of mixture effects in combination with other methods that allow robust quantification of effects of substances. 

From a technical perspective, benefits of the proposed workflow compared to a LC-MS assay only is the rapid and efficient isolation of peptides of interest from a complex sample. Peptide responses are improved and ion suppression effects avoided while reducing required chromatographic separation times in parallel. In general, sensitivity is improved and sample turnover increased (Weiß, 2015[29]; Weiß et al., 2015[31]). Moreover, additives like detergents which are required for protein and especially membrane protein extraction are highly efficiently removed from the sample, which makes the sample compatible with LC-MS read out (Eisen et al., 2013[8]; Weiß et al., 2014[32], 2015[31]). Hence, the method is highly suitable for the quantification of challenging proteins like transporters, which are hard to quantify on intact protein level. Additionally, the assays' sensitivity can be adjusted to the analyte concentration by adapting sample, standard, antibody and bead amounts (Weiß, 2015[29]).

The comparison of available enzymatic and mRNA data with the protein abundance data in this study revealed that for some of the data mRNA, protein and enzyme activity correlate quite well, while for others like CYP1A1 the correlation is not equally high. Reasons for this may be differences in turnover of biomolecules like mRNA and protein. A combination of omics methods to better substantiate the understanding of the hepatotoxicity of azoles has also been discussed before (Seeger et al., 2019[26]). This underlines the need to take into account more sorts of biomolecules when analyzing toxicity rather than one.

To substantiate regulatory conclusions, it is recommended that data obtained at the mRNA level should be confirmed by using proteomic or enzymatic method (Gautier et al., 2016[9]). Even though protein levels on their own would not be used in regulation of chemical substances for purposes such as classification and labeling or derivation of reference values, they are very important for elucidating a mode or mechanism of action (Gautier et al., 2016[9]). Hence, the assays developed here are suitable to be used for the analysis of mixture effects in combination with other methods that allow robust quantification of effects of substances. 

## Conflict of interest

The authors declare no conflict of interest.

## Supplementary Material

Supplementary tables

Supplementary figure 1
