# Supplementary material for: Induction and repression effects on CYP and transporter protein abundance by azole mixture uptake in rat liver
Source: EXCLI J. 2020 Jun 26;19:904–16. doi: 10.17179/excli2020-2311 (PMC7744963; doi:10.17179/excli2020-2311)
Supplement: Supplementary figure 1 [file EXCLI-19-904-s-002.pdf]

EXCLI Journal 2020;19:904-916 – ISSN 1611-2156
Received: April, 20, 2020, accepted: June 19, 2020, published: June 26, 2020

Original article:

INDUCTION AND REPRESSION EFFECTS ON CYP AND TRANSPORTER PROTEIN ABUNDANCE BY AZOLE MIXTURE UPTAKE IN RAT LIVER

Helen Hammer[1,3], Flavia Schmidt[2], Tanja Heise[2], Constanze Knebel[2], Alexander Dabrowski[2], Hannes Planatscher[1,3], Carsten Kneuer[2], Philip Marx-Stoelting[2], Oliver Pötz[1,3,*]

[1] NMI Natural and Medical Sciences Institute at the University of Tübingen, Reutlingen, Germany
[2] BfR, Department of Pesticides Safety, Max-Dohrn-Str. 8-10, 10589 Berlin, Germany
[3] SIGNATOPE GmbH, Reutlingen, Germany

* **Corresponding author:** Oliver Pötz, NMI Natural and Medical Sciences Institute at the University of Tübingen, Markwiesenstrasse 55, 72770 Reutlingen, Germany, E-mail: poetz@nmi.de

http://dx.doi.org/10.17179/excli2020-2311

This is an Open Access article distributed under the terms of the Creative Commons Attribution License (http://creativecommons.org/licenses/by/4.0/).

EXCLI Journal 2020;19:904-916 – ISSN 1611-2156
Received: April, 20, 2020, accepted: June 19, 2020, published: June 26, 2020

motif	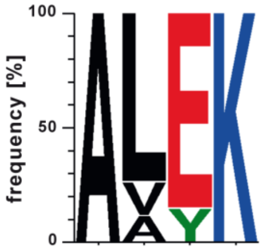	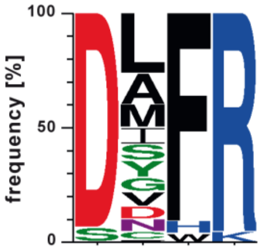	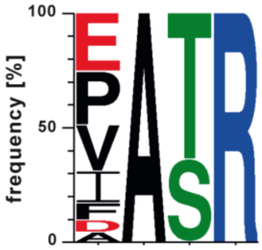
antigen	A L E K	D L F R	E A T R
target	SLC10A1	SLC22A7, -A8	CYP2E1
#peptides/ tags	36 / 4	90 / 15	69 / 11
peptide ratio	8.6%	20.4%	11.8%
motif	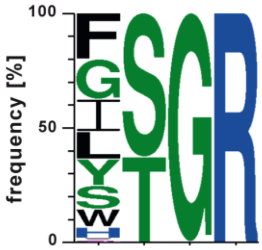	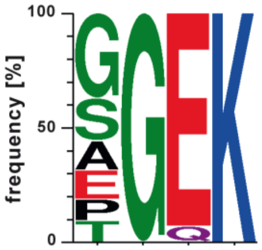	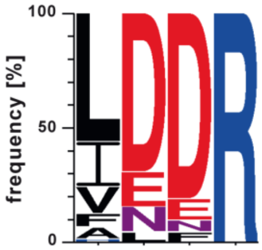
antigen	F S G R	G G E K	L D D R
target	CYP2B3, -2C11 -2C12, -2C13, -2C55	ABCC3	CYP2B2
#peptides/ tags	195 / 21	61 / 7	126 / 17
peptide ratio	21.1%	17.7%	33.3%
motif	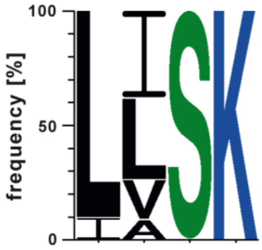	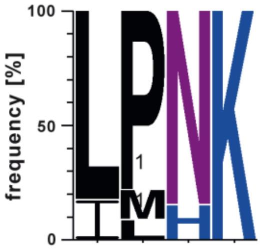	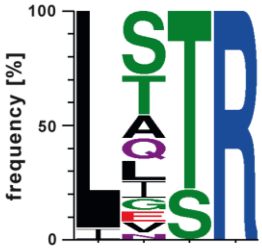
antigen	L I S K	L P N K	L T T R
target	CYP1A1, -1A2	CYP3A9	ABCB1, -B1a, -B11
#peptides/ tags	90 /5	21 / 5	122 / 13
peptide ratio	22.6%	20%	10.8%
motif	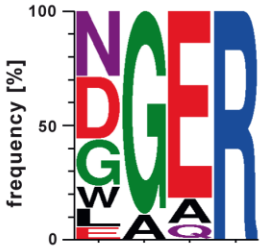	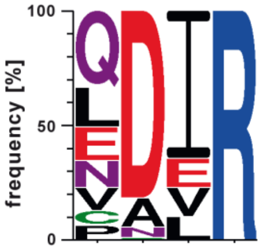	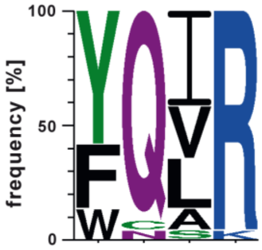
antigen	N G E R	Q D I R	Y Q V R
target	CYP2B1, -2B2	ABCB1, -B1a	ABCC2
#peptides/ tags	49 / 9	97 / 16	71 / 13
peptide ratio	9.1%	20.0%	29.2%

EXCLI Journal 2020;19:904-916 – ISSN 1611-2156
Received: April, 20, 2020, accepted: June 19, 2020, published: June 26, 2020

Supplementary Figure 1: Characterization of TXP antibody binding motifs. All used TXP antibodies were characterized as described in detail for the anti-FTNR antibody in **Fehler! Verweisquelle konnte nicht gefunden werden.**. Immunoprecipitation of 20 µg proteolyzed human cell line blend containing equal amounts of HepG2, HEK 293 and HCT116 was performed with each TXP antibody. The precipitated peptides were identified via non-targeted MS analysis. All c-termini which were enriched significantly by the immunoprecipitation were included in the motif. The antigen and intended target proteins are listed as well as the number of identified peptides and c-terminal tags the motif is based on. The peptide ratio describes the number of peptides containing the target antigen which were precipitated in comparison to the number of peptides determined by in-silico digest.
